# Erythropoietin in Glaucoma: From Mechanism to Therapy

**DOI:** 10.3390/ijms24032985

**Published:** 2023-02-03

**Authors:** Yi-Fen Lai, Ting-Yi Lin, Yi-Hao Chen, Da-Wen Lu

**Affiliations:** Department of Ophthalmology, Tri-Service General Hospital, National Defense Medical Center, Taipei 11490, Taiwan

**Keywords:** erythropoietin, neuroprotection, retinal ganglion cell, glaucoma

## Abstract

Glaucoma can cause irreversible vision loss and is the second leading cause of blindness worldwide. The disease mechanism is complex and various factors have been implicated in its pathogenesis, including ischemia, excessive oxidative stress, neurotropic factor deprivation, and neuron excitotoxicity. Erythropoietin (EPO) is a hormone that induces erythropoiesis in response to hypoxia. However, studies have shown that EPO also has neuroprotective effects and may be useful for rescuing apoptotic retinal ganglion cells in glaucoma. This article explores the relationship between EPO and glaucoma and summarizes preclinical experiments that have used EPO to treat glaucoma, with an aim to provide a different perspective from the current view that glaucoma is incurable.

## 1. Introduction

Glaucoma is the second leading cause of blindness worldwide, and in 2020, an estimated 11.2 million people became blind as a result of glaucoma [1]. In the early stage, glaucoma is largely asymptomatic, and visual-field defects only become obvious in the late stage of the disease. Thus, the number affected may be much higher. Glaucoma is a group of heterogeneous optic neuropathies characterized with progressive ganglion-cell death, progressive optic nerve axon degeneration, and corresponding visual-field defects [2]. Clinically, there are an increase in the optic-disc cup-to-disc ratio, optic-disc hemorrhage, and increased intraocular pressure (IOP). The reason for the increased IOP is unclear, but lowering IOP can slow the progression of glaucoma. However, in a small number of patients, lowering IOP does not alter the clinical course, suggesting that the pathological mechanism involves something besides IOP-induced ganglion-cell damage. Studies have reported that glaucoma likely has a complex, multicausal pathological mechanism that may involve ischemia, excessive oxidative stress, neuroinflammation, loss of neurotropic factors, and neuron excitotoxicity: factors that share the same pathophysiology with many neurodegenerative diseases [3]. As a consequence, some scientists choose neuroprotection as a treatment strategy for glaucoma.

Erythropoietin (EPO) is traditionally considered as an oxygen-regulating hormone for erythropoiesis. In hypoxia conditions, hypoxia-inducible factors (HIFs) upregulate EPO production. Aside from well-known physiological functions, EPO is found to exert innate repair effects via binding to its heterogeneous receptor. The heterogeneous receptor has been detected in extrahematopoietic tissues, such as the heart, the central nervous system, and even the retina [4,5,6]. EPO secreted by ganglion cells and retinal pigmented epithelium in paracrine signaling could target the EPO receptor (EPOR) via photoreceptors, bipolar cells, and amacrine cells. Previous studies reported EPOR upregulated in retinal ischemia [7]. Our previous study also found that exogenous EPO could attenuate N-methyl-D-aspartate (NMDA)-mediated retinal-ganglion-cell (RGC) damage [8,9]. Many studies have also proven that EPO has antiapoptotic, anti-inflammatory, and antioxidative effects in prevention of RGC damage. Therefore, we summarized the relationship between EPO and glaucoma and comprehensively reviewed research related to EPO in glaucoma treatment.

## 2. Pathogenesis of Glaucoma

Glaucoma is characterized with optic-disc cupping, neuroretinal-rim thinning, and optic-disc pallor, which represent a loss of ganglion-cell bodies and their axons. Various insults on retinal ganglion-cell bodies and axons, irrespective of the initial site, can cause glaucoma [10]. Glaucoma is thought to be caused by complex interactions among these insults rather than any one of them functioning individually.

### 2.1. Intraocular-Pressure-Related Retinal Ganglion-Cell Apoptosis

Many studies have indicated that axon degeneration precedes RGC-soma death in glaucoma [11]. Increased IOP impedes axoplasmic flow at the lamina cribrosa. This axon-transport interruption deprives RGCs of retrograde brain-derived neurotrophic factor (BDNF), which induces secondary apoptosis. Some studies have reported that elevated IOP increased secretion of matrix metallopeptidase-9 (MMP-9) and degraded laminin [10,12], which reduced cell to extracellular matrix (ECM) communication, leading to RGC apoptosis [13]. Elevated IOP also upregulated the expression of tumor necrosis factor-alpha (TNF-α) in astrocytes [12,14], which in turn triggered nitric oxide synthase (NOS)-2 [14], resulting in RGC apoptosis under oxidative stress.

### 2.2. Decreased Ocular Perfusion

Glaucoma and insufficient blood perfusion are positively correlated [15], and some have suggested that glaucoma is a chronic anterior ischemic optic neuropathy. One study showed that migraines were more common among patients with normal-tension glaucoma than in healthy controls [16]. Patients with glaucoma have higher rates of diffuse cerebral small-vessel ischemia seen in magnetic resonance imaging (MRI) [17]. Nocturnal hypotension also accelerates progression of glaucoma [18]. Plasma and aqueous humor levels of endothelin-1 (ET-1), a vasoconstrictor that binds to receptors on smooth muscle cells and pericytes to constrict blood vessels, are increased in patients with normotensive glaucoma [19,20,21], confirming abnormal vascular function as an important factor in the pathophysiology of glaucoma [15]. ET-1 binding to its subtype receptor, ETb, increases nitric oxide (NO) release [22]. This NO may stimulate vascular dilation and remove ET-1 from circulation [23]. Vasospasm followed by vasodilation causes ischemia–reperfusion, increasing oxidative stress and causing RGC death. Although NO seems to be deleterious to RGCs, some studies have reported that the NO-soluble guanylyl cyclase (sGC)-cyclic guanosine monophosphate(cGMP) pathway demonstrated an antiapoptosis effect in retinal cells via Akt phosphorylation [24]. In one animal study, cGMP protected retinal cells against ischemic insults [25]. cGMP also prevents RGC loss via relaxation of trabecular meshwork, lowered IOP and raised blood flow [26]. Dysfunction in the NO/sGC/cGMP signaling pathway disturbs the balance of ocular blood flow and is linked to RGC loss [27].

### 2.3. Glutamate Excitotoxicity

Glutamate is an important neurotransmitter in the retina; it opens calcium ion channels through NMDA receptors or α-amino-3-hydroxy-5-methyl-4-isoxazolepropionic acid (AMPA) receptors [28]. When these channels open, extracellular calcium flows into neurons, where it functions as a second messenger to initiate neurotoxic signal pathways via the μ-calpain/Bax/cytochrome c/caspase-9 axis [9]. To avoid glutamate-induced neurotoxicity, Müller cells and astrocytes express glutamate transporters that transfer glutamate into the cell, where it is converted to nontoxic glutamine. Neurons reabsorb glutamine and convert it to glutamate to complete the glutamate/glutamine cycle [29]. Glutamate-induced toxicity was first recognized in 1957 [30]. In animal experiments, mice with glaucoma had decreased expression of glutamate transporters [31], which increased extracellular glutamate. Injection of glutamate in mice caused severe neuronal cell degeneration [30]. Intraocular injection of glutamate enlarged the cupping of optic discs in neonatal mice [31]. Intravitreal injection of glutamate in adult mice caused RGC death [32]. However, the results of glutamate excitotoxicity studies in monkeys and humans are controversial. Dreyer et al. reported that the concentration of glutamate in the vitreouses of monkeys with glaucoma was eight times higher than that in normal monkeys [33]. However, subsequent studies were not able to replicate Dreyer’s findings. Others reported that the glutamate concentration in the vitreouses of patients and monkeys with glaucoma was not different from that in controls [34,35]. Although some published data by Dreyer et al. has been discredited, a wealth of research on the topic supports a role for glutamate toxicity in the retina. Based on others’ research, we concluded that glutamate can indeed cause RGC damage.

### 2.4. Oxidative Stress

Glaucoma can be induced via increased oxidative stress that arises from inflammation, hypoxia, ischemia, and mitochondrial dysfunction [36]. Oxidative stress can indirectly or directly damage RGCs. Oxidative stress indirectly affects the adhesion of trabecular meshwork cells to the ECM, changes the cytoskeletal structure of trabecular meshwork cells, transforms trabecular meshwork cells into myoepithelial cells, reduces ECM breakdown, reduces aqueous humor outflow, and increases IOP, causing a pathological cascade that leads to glaucoma [37,38]. Oxidative DNA damage in trabecular meshwork cells was more severe in patients with glaucoma than in controls without glaucoma [39], and this oxidative stress was positively correlated with degree of visual-field damage [40]. Oxidative stress can also directly damage RGCs [41,42,43]. Exogenous reactive oxygen species (ROSs) can induce apoptosis of immortalized ganglion cells via a caspase-independent pathway [44], and reducing ROSs can prevent apoptosis of RGCs.

### 2.5. Neurotrophic-Factor Deprivation

Brain-derived neurotrophic factor is essential for neuronal function and regeneration [45]. It is secreted into synaptic clefts and binds to tropomyosin receptor kinase B (TrkB) on the presynaptic cell surface. The BDNF-TrkB complex is then internalized via endocytosis and transported to the cell body of the presynaptic neuron. BDNF-TrkB signaling modulates formation of dendrites and synapses through the mitogen-activated protein kinase (MAPK), phosphatidylinositol 3-kinase (PI3K), and phospholipase C-γ (PLC-γ) pathways [46,47]. The RGC axons of rats were abnormally swollen in acute glaucoma. Labeling of TrkB at the optic nerve head was increased, and radiolabeling of BDNF in the RGC layer of mice was half that of the controls [48,49]. These findings, TrkB-receptor accumulation and blocked retrograde transport of BDNF from the superior colliculus to the optic nerve head, suggest that BDNF deprivation might contribute to RGC apoptosis in glaucoma.

## 3. Neuroprotection Effect of Erythropoietin

EPO exerts pleiotropic effects via binding to different receptor subtypes. Binding to the homogenous isoform EPOR2 mainly stimulates the maturation and differentiation of erythrocyte precursors, whereas binding to EPOR/BcR, the heterogeneous isoform with eight-to-sixteenfold weaker affinities, can induce neuroprotection [50]. The heterodimeric receptor has been identified in RGCs, inner nuclear layers, and photoreceptors and implicated in most of their antiapoptotic [51], anti-inflammatory [52], and antioxidative effects [52] in the ocular disease. Through EPOR/BcR binding, EPO can initiate Wingless (Wnt) signaling, which is responsible for cell differentiation and survival [53]. Binding of EPO to EPOR can induce Janus kinase 2 (JAK2) phosphorylation and subsequently activate the STAT3/5, MAPK, PI3K/Akt, and nuclear factor kappa-light-chain-enhancer (NF-κB) pathways. The last molecules in the STAT3/5 and MAPK pathways translocate into the nucleus and upregulate antiapoptotic proteins Bcl-2 and Bcl-XL to inhibit apoptosis [54]. EPO also activates the PI3k/Akt pathway, which overlaps with the NO/sGC/cGMP pathway and exhibits survival-promoting properties in retinal neurons [24]. EPO also inhibits cytochrome-c leakage from mitochondria [55], inhibits caspase-9 activation, and prevents DNA fragmentation [56]. EPO can also activate the NF-κB pathway, which exerts protective effects via caspase-activity blocking, suppression of TNF-α–related apoptosis, direct enhancement of activation of Bcl-XL, and removal of cellular ROS [57,58]. EPO also has anti-inflammatory activity. It can reduce production of inflammatory cytokines, including ICAM-1, IL-1β, IL-6, and TNF-α, and increase production of the anti-inflammatory cytokine IL-10 [59]. EPO can also facilitate immunomodulation via stimulation of regulatory T-cell proliferation and inhibition of conventional T-cell proliferation [60]. Moreover, EPO has been shown to attenuate oxidative stress. It has induced heme oxygenase-1 to protect astrocytes via the PI3k/Akt pathway [61,62]. EPO has also increased the expression of glutathione peroxidase, a powerful antioxidant, to reduce ROS toxicity [63]. Therefore, EPOR/BcR has been named as a tissue-protective receptor and an innate repair receptor [64].

**Figure 1:**
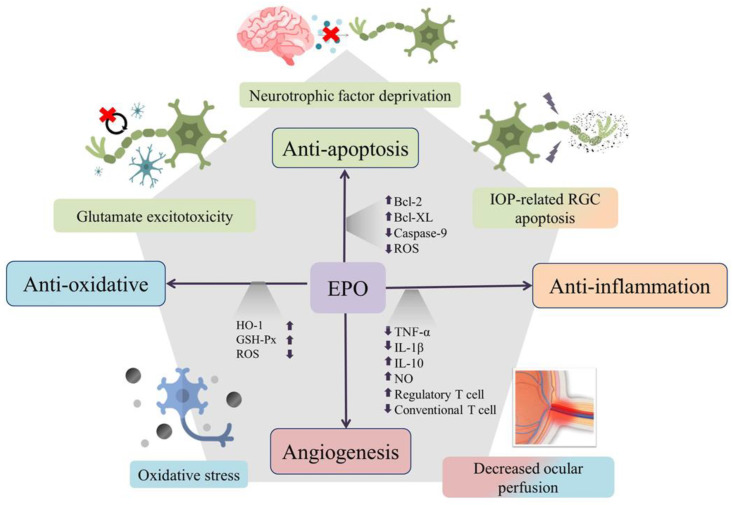
Erythropoietin (EPO) is neuroprotective through its antiapoptotic, antioxidative, and anti-inflammatory effects. The multifunctionality of EPO is used to target the multicausal mechanism of glaucoma. Through upregulation of antiapoptotic proteins Bcl-2 and Bcl-XL, inhibition of caspase-9 activation, and removal of reactive oxygen species (ROS), EPO can exert its antiapoptosis action against intraocular-pressure-related retinal ganglion-cell injury, glutamate excitotoxicity, and damage from neurotrophin factor deprivation. EPO can also increase levels of heme oxygenase-1 (HO-1) and glutathione peroxidase (GSH-Px) to reduce oxidative stress and can promote immunomodulation through decreased inflammatory cytokines, increased anti-inflammatory cytokines and enhancement of regulatory T-cell proliferation. Theoretically, the angiogenesis and antioxidative effects of EPO might also contribute some protective effects against injury from decreased ocular perfusion in glaucoma.

Many studies have demonstrated that EPO production is associated with glaucoma. One study reported that the EPO concentration in the aqueous humor is increased in eyes with primary open-angle glaucoma, pseudoexfoliation glaucoma, and neovascular glaucoma [56,65]. Other studies revealed that there is a significant increase in aqueous EPO levels in primary open-angle glaucoma (POAG) patients compared with in cataract patients, but there is no difference in the plasma EPO level between POAG and cataract patients [66]. The aqueous EPO level is proportionate to the level of IOP in eyes with pseudoexfoliation glaucoma [65]. EPO-level increase in glaucoma is considered as a compensatory neuroprotective action secondary to glaucomatous damage. Based on the experimental results above and the molecular pathway of EPO, administration of EPO might have therapeutic potential in treating glaucoma (see Figure 1). Below, we summarize the latest studies about EPO for glaucoma.

## 4. Studies of Erythropoietin for Glaucomatous Optic Neuropathy

### 4.1. Exogenous Erythropoietin for Glaucoma

Because of its complex pathogenesis, no single animal model can perfectly recapitulate the pathogenic mechanism of glaucoma [67]. Thus, the efficacy of EPO has been tested in different animal models (Table 1). Some animal models have simulated IOP elevation to induce ocular ischemia and interrupt retrograde axonal transport, including anterior chamber cannulation, episcleral-vessel cautery, and intracameral injection of microbeads [67]. In these models, the expression levels of EPO and EPOR increased after long-term elevation of IOP, proving that EPO/EPOR signaling is an important protective signal against IOP elevation [68,69]. Either intravitreal injection or intraperitoneal administration of EPO to amplify this intrinsic EPO/EPOR signal could increase RGC survival [68,70]. In one study, the EPO-treated group had thicker retinas than did the control group [71]. Aside from morphological preservation, exogenous EPO also promoted functional recovery, as demonstrated with electroretinograms (ERGs) [72]. In rats with acute IOP elevation, the ERG b wave was significantly reduced. Recovery was improved in EPO-treated rats compared with untreated controls [7,71]. Exogenous EPO diminished terminal deoxynucleotidyl transferase dUTP nick-end labeling (TUNEL) in the ischemic retina, suggesting its antiapoptotic properties [7].

In an animal model of oligemia induced via bilateral common carotid artery occlusion, the chronic cerebral ischemia mimicked decreased ocular perfusion. In rats with cerebral ischemia, the latency and amplitude of the P1 wave were, respectively, increased and decreased, and intranasal delivery of recombinant EPO significantly restored visual function [73]. In a model of glutamate-induced retinal toxicity, pretreatment with EPO protected RGCs from glutamate- and nitric-oxide-induced toxicity via upregulation of Bcl-2 [74]. Inhibition of signaling via STAT-5, MAPK/ERK, and PI3k/Akt blocked the protective effect of EPO [8]. Coadministration of EPO and NMDA decreased proapoptosis signaling via downregulation of μ-calpain, Bax, and caspase-9 [8,9]. DBA/2J mice, which spontaneously develop glaucoma, are a well-characterized model of pigmentary glaucoma [80]. Administration of EPO to DBA/2J mice promoted RGC survival. The protective effect of EPO is similar to that of a known neuroprotective agent, NMDA-receptor antagonist memantine [75]. In animal models of optic nerve transection and optic nerve crush, axotomized RGCs underwent axon degeneration, which mimics axonal degeneration prior to soma apoptosis in glaucoma. Degenerated axons induce secondary soma apoptosis via activation of capspase-3 and -9 [81] and downregulation of the RAS/RAF/ERK and PI3k/Akt pathways [82]. EPO promoted the survival of axotomized RGCs through activation of ERK-1/-2 and PI3k/Akt signaling [76,77]. In addition to its neuroprotection effect, EPO also promoted axon regeneration in rats following optic nerve crush and optic nerve transection with peripheral nerve grafting [78].

Although the studies above confirmed the neuroprotective effects of EPO, many limitations to its practical application remain. For example, EPO has a short half-life (t_1/2_~5.6 h), and its affinity for EPOR2 is much higher than its affinity for EPOR/BcR; thus, side effects, such as polycythemia and thrombosis, can occur. For these reasons, researchers have altered the structure of EPO to increase its half-life and reduce side effects. Rex et al. developed a novel EPO derivative via changing arginine 76 to glutamate (EPOR76E), rendering it nonerythropoietic but still neuroprotective. That team encapsulated a His-tagged version of this EPO derivative in either poly (lactic-co-glycolic acid) (PLGA) or poly (propylene sulfide) (PPS) microparticles to prolong its release [79]. They injected these two EPO-containing microparticles into the vitreous bodies of mice in a microbead model and found that both PLGA-EPOR76E and PPS-EPOR76E preserved axon function and prevented axon degeneration via enhancement of antioxidant capacity. PPS-based microparticles are especially promising and safe. This strategy eliminates the limitations of EPO use, but the safety and feasibility of PPS-EPOR76E need to be examined in additional studies.

### 4.2. Endogenous Erythropoietin for Glaucoma

EPO has the disadvantages of a short half-life and erythropoiesis induction, which limit its clinical application. To induce a longer, sustained, and endogenous treatment effect for glaucoma, some scientists have focused on gene therapy (Table 2). EPO gene therapy has been shown to be neuroprotective in models of nerve damage and neurodegenerative disease. For example, injection of a herpes-simplex-virus (HSV)-based vector that encoded EPO into a model of spinal-cord injury minimized injury size, preserved large-caliber axons, and promoted synaptogenesis [83]. Adeno-associated viral serotype 9 (AAV9)-mediated EPO gene delivery into the striata of rats protected nigral dopaminergic neurons against 6-hydroxydopamine (6-OHDA)-induced toxicity and improved the behavioral performance in a rat model of Parkinson’s disease [84].

HSV, AAV, and lentivirus vectors have all been successfully used to deliver therapeutic genes [85,86,87]. AAV is the most popular, since its genome composition and mechanisms of DNA replication and transcription, virion assembly, the transduction pathway, and poor immunogenic response are well-characterized [88]. Advances in the knowledge of AAV and virus–host interaction have made AAV a practical vector for gene therapy [89]. Recombinant AAV vectors were generated via combination of the genome of one serotype with the capsid of another type. The hybrid virion had capsid type-dependent tropism, allowing for efficient transgene delivery to specific organs [79]. Among the different AAV serotype vectors, AAV2, 4, 5, and 8 are specifically for retinal tissue [90]. Recombinant AAV gene-therapy products are commercially available for treatment of ophthalmic diseases. In 2017, voretigene neparvovec-rzyl (Luxturna), which uses an AAV2 vector, was approved by the United States Food and Drug Administration to treat patients with biallelic RPE65 mutation-associated retinal dystrophy [91]. The development of voretigene neparvovec-rzyl suggests the potential of gene therapy for glaucoma.

Rex et al., administered recombinant AAVs, which carried EPO (rAAV2/5.CMV.EPO) or EPOR76E (rAAV2/5.CMV.EPOR76E), via intramuscular injection into the quadriceps of 1-month-old DBA/2J mice [92]. Profound axon degeneration and gliosis of optic nerves were observed in control mice (rAAV2/5.CMV.eGFP), whereas mice treated with rAAV2/5.CMV.EPO or rAAV2/5.CMV.EPOR76E had little or no axon degeneration at 10 months of age. In addition to anatomical preservation, visual function was also preserved in mice treated with either EPO vector based on flash visual evoked potential (fVEP). In mice administered with either wild-type EPO or EPO76E, wild-type EPO significantly increased hematocrit levels, whereas EPO76E only slightly increased hematocrit levels. That study group then tested the treatment efficacy of EPOR76E gene therapy in two different glaucomatous animal models: DBA/2J mice and the murine microbead occlusion model [93]. In the murine microbead model, which simulated closed-angle glaucoma without overt neuroinflammation, they administrated rAAV.EPOR76E at two timings: prior to the onset of elevated IOP (i.e., pre-IOP) and at the onset of elevated IOP (i.e., post-IOP). They tested the efficacy of rAAV.EPOR76E at 4 weeks after microbead injection, which simulated early-stage glaucoma, and found that rAAV.EPOR76E preserved RGC axons and their antegrade transport not only before IOP elevation but also after IOP elevation. In the DBA/2J model with anterior segment synechiae, pigment dispersion, and neuroinflammation, those researchers assessed the therapeutic effect of EPOR76E at 10 months of age, which represented late-stage glaucoma with severe RGC loss. In their previous study, they treated DBA/2J mice with rAAV.EPOR76E at 1 month of age and observed complete neuroprotection of RGCs, axons, and VEP at 10 months of age. However, in the follow-up study, the level of protection at 10 months of age was much lower, suggesting that late administration of EPO76E could not completely stop disease progression once it began. The same study group then treated 5-month-old DBA/2J mice with either rAAV.EPOR76E or the control vector to determine if modulation of neuroinflammation and oxidative stress plays a vital role in the neuroprotective effect of EPOR76E gene therapy [94]. As expected, administration of the EPOR76E vector preserved axon transport and VEP. The number of microglia decreased but proliferation was unaffected. Levels of proinflammatory cytokines IL-1, IL-12, IL-13, IL-17, CCL4, and CCL5 decreased in the retinas of EPOR76E-treated mice. Antioxidant enzyme expression was also increased in treated mice. Those study results showed that rAAV.EPOR76E preserves axons and RGC function via attenuation of neuroinflammation and oxidative stress.

Despite these promising results in animal models, many safety concerns need to be addressed before clinical application. For systemically secreted proteins such as EPO, precise regulation of gene expression in normal and unhealthy ganglion cells is needed, as dysregulation of EPO expression could cause lethal adverse events, such as polycythemia or thromboembolic events. Utilizing a promoter that is specifically activated during RGC stress is one strategy for expression regulation. One study used the monocyte chemoattractant protein-1 (Mcp-1) promoter, which is activated in stressed RGCs [95]. Although the method targets stressed RGCs, there is no way to regulate this expression once the transduced cells are switched on. Another strategy for controlling transgene expression is inducible promoter systems, such as tetracycline, rapamycin, and morpholino-regulated hammerhead ribozyme [96,97]. Hine-Beard et al. injected a rAAV vector with EPOR67E, under the control of a tetracycline-inducible promoter, into the subretinal space of homozygous retinal degeneration slow (rds/rds) mice [96]. Then, mice that were fed doxycycline water were compared with mice that were administered a single intraperitoneal injection of doxycycline and with controls. EPOR67E levels were higher in mice that were fed doxycycline water than in doxycycline-injected mice. The outer nuclear layer was thicker in the retinas of mice that were fed doxycycline water than in the control groups. Therefore, transcription factors responded to the orally administered drug to regulate transgene expression. However, experimentation regarding an inducible promoter system in glaucoma animal models is still lacking.

### 4.3. Other Novel Uses of Erythropoietin for Glaucoma

In late-stage glaucomatous optic neuropathy, mass death of RGCs is the leading cause of blindness. The only way to restore visual function is transplantation of embryonic or induced pluripotent stem cells to replace degenerated RGCs with functional RGCs [98]. However, the various subtypes of RGCs complicate development of transplantable RGCs and their functional integration. In addition, the projections of RGC axons have target and retinotopic specificity [99]. Although most RGC axons project to the lateral geniculate body, others project to corresponding areas in the brain, such as the suprachiasmatic nucleus and the superior colliculus. RGCs in the retina also have corresponding visual fields in the brain. Formation of synapses between RGC axons and distant targets shapes the visual information map, enriching spatial-sensation accuracy. Therefore, considerable development is needed before application of embryonic or induced pluripotent stem cell therapy to replace degenerative RCGs. Current therapeutics focus on neuroprotection to delay or avoid cell death and retain viable RGCs.

Mesenchymal stem cells (MSCs) are multipotent adult stem cells that can be differentiated into several cell types, including adipogenic, chondrogenic, osteogenic, and even retinal progenitor cells [100,101]. They exert immunomodulatory effects [92,102] through secretion of exosomes and can produce various neurotrophic and growth factors to nourish damaged retina tissues [103,104,105]. They can promote RGC survival and axon regeneration. Additionally, they can be isolated from different tissues using less-invasive procedures, expanded in vitro for further gene editing, and autologously transplanted to avoid rejection. Thus, MSCs have potential as therapeutics for glaucomatous optic neuropathy. Many preclinical studies have shown that MSC-mediated neuroprotection is safe and effective in animal models of glaucoma [106,107,108]. However, transplanted MSCs must survive in pathological environments before cell programming can occur. Some investigators incorporated the EPO gene into MSCs to enhance engraftment [109,110]. Guan et al. compared the effects of MSCs; EPO-gene-modified MSCs; and doxycycline-inducible, EPO-expressing MSCs in a rat model of retinal degeneration. Rats transplanted with either EPO-gene-modified MSCs or doxycycline-inducible, EPO-expressing MSCs showed greater improvement in retinal morphology and function [111]. Ding et al. also modified human MSCs derived from Wharton’s jelly (hWJMSCs), with lentivirus particles that encoded the EPO gene [112]. EPO concentration was significantly increased in the conditioned medium collected from EPO-transduced MSCs (EPO-MSCs) compared with that collected from nontransduced MSCs. The human retinoblastoma was exposed to a toxic dose of glutamate and then incubated with supernatant from EPO-MSCs. Those researchers observed reduced mitochondrial depolarization and increased retinal neuron survival during coculture with EPO-MSCs. Kim et al. assessed the protective effects of both EPO-MSCs and MSCs treated with 10 IU of EPO (10U-MSCs) against H_2_O_2_-induced oxidative stress and staurosporine-induced apoptosis [113]. They found that EPO-MSCs have a higher protection rate and higher levels of intracellular ERK1/2 signaling than do 10U-MSCs. That study proved that MSCs transduced with the EPO gene have greater synergistic effects on neurotrophic factor production than do MSCs cocultured with EPO. Moreover, MSCs can be used to deliver EPO systemically because they have homing properties and can cross the brain–retinal barrier. This phenomenon highlights the synergic relationship between MSCs and EPO. Thus, incorporating the EPO gene into stem cells could magnify the therapeutic effects of MSC therapy and avoid the side effects of intraocular injection, making it a promising treatment for glaucomatous optic neuropathy.

## 5. Conclusions

Over the next decade, a better understanding of the pathophysiology of glaucoma will provide new insights for development of novel therapeutics. An ideal therapeutic should have multiple activities that target the multifactorial pathogenic mechanism of glaucoma. The antiapoptotic, anti-inflammatory, and antioxidative effects of EPO make it a promising therapeutic for treatment of glaucoma. Whether that therapeutic is EPO or another candidate, there is an urgent need to preserve the vision of the many people with this sight-threatening disease. We foresee future studies that address EPO with better-controlled regulation to magnify its therapeutic effects in glaucoma.

## Figures and Tables

**Table 1:** Summary of studies that have evaluated erythropoietin for treatment of glaucoma.

Authors	Year	Study Design	Number of Animals	Intervention	Main Outcomes
Chang et al. [8]	2013	Intervention study	RGCs isolated from adult Wistar rats	RGC coculture with NMDA, 500 µM: (1) + EPO 100 ug/mL; (2) + EPO 100 ug/mL + PD98059 (MAPK/ERK inhibitor) 20 µM; (3) + EPO 100 ug/mL + Wortmannin (PI3K/Akt inhibitor) 1 µM; (4) + EPO 100 ug/mL + STAT5 inhibitor 200 µM.	(1) EPO improved RGC survival under neuron excitotoxicity. (2) Inhibitors of STAT-5, MAPK/ERK, and PI3K/Akt impaired the protective effect of EPO.
Cheng et al. [9]	2020	Randomized intervention study	Total: 125 Wistar rats	Randomly assigned into five groups: (1) Control; (2) Intravitreal NMDA80; (3) Intravitreal NMDA80 + 10 ng EPO; (4) Intravitreal NMDA80 + 50 ng EPO; (5) Intravitreal NMDA80 + 250 ng EPO.	EPO protected RGCs and bipolar cell-axon terminals in IPL through downregulation of apoptotic factors to attenuate NMDA-mediated excitotoxic retinal damage.
Fu et al. [68]	2008	Comparative study	Not mentioned	(1) Argon laser coagulation of the episcleral and limbal veins to induce chronic hypertension: -IVI EPO (2U); -IVI PBS; -IVI sEPOR (20 ng). (2) Optic nerve transection: -I.P. EPO 5000 U/kg; -I.P Saline.	(1) Müller cells were the main source of EPO in the normal retina. (2) Expression of EPO and EPOR was increased after ocular hypertension. (3) Soluble EPOR exacerbated ocular hypertensive injury. (4) IVI EPO rescued RGCs after chronic ocular hypertension, while I.P. EPO rescued RGCs in a model of optic nerve transection.
Zhong et al. [69]	2008	Comparative study	Total: 75 Sprague Dawley rats	Assigned to five groups: (1) Untouched control; (2) Sham-operated group: anterior chamber cannulation (IOP raised to 15 mmHg for 60 min); (3) Anterior chamber cannulation (IOP raised to 70 mmHg for 60 min); (4) Anterior chamber cannulation (IOP raised to 70 mmHg for 60 min) + retrobulbar EPO (1000 U); (5) Anterior chamber cannulation (IOP raised to 70 mmHg for 60 min) + retrobulbar vehicle.	(1) RGC densities: Sham-operated group > IOP elevation + EPO group > IOP elevation + vehicle group. (2) EPO/EPOR densities: IOP elevation + EPO group > IOP elevation + vehicle group or IOP elevation.
Tsai et al. [70]	2005	Comparative study	Total: 29 Sprague Dawley rats	Assigned to four groups: (1) Unoperated control; (2) EVC only; (3) EVC + IVI saline; (4) EVC + IVI EPO (200 ng).	A single intravitreal 200 ng dose of EPO appeared to have a protective effect on RGC viability in an in vivo rat model of glaucoma.
Resende et al. [71]	2018	Comparative study	Total: 26 Wistar Hanno-ver albino rats with unilateral glaucoma induced through coagulation of three episcleral veins in the right eye. Case (right eye): 13 eyes. Control (left eye): 13 eyes.	Subconjunctival injection of 1000 IU EPO versus placebo	EPO improved both scotopic and photopic amplitude. Retinal thickness was thicker in the EPO group.
Jehle et al. [72]	2010	Comparative study	(1) IOP elevation, *n* = 9–21 (2) Optic nerve compression, *n* = 6–8	(1) Vitreous cannulation (IOP raised to 120 mmHg for 55 min: -IVI EPO (2 U); -IVI EPO (20 U). (2) Optic nerve compression for 10 s: -IVI EPO (2 U); -IVI EPO (20 U).	With 20U EPO, postischemic function was increased via ERG and VEP.
Zhou et al. [73]	2020	Comparative study	Total: 60 Sprague Dawley rats	Assigned to three groups: (1) Sham-operated; (2) Bilateral common carotid artery occlusion (2VO)-induced chronic cerebral ischemia + intranasal saline; (3) 2VO + intranasal EPO (150 U/120 ul).	(1) The latency and amplitude of the P1 wave in fVEP were significantly increased and decreased, respectively, in the sham-operated group and the 2VO + saline group. (2) EPO treatment markedly reduced the latency and elevated the amplitude of the P1 wave compared with in the sham-operated group or the 2VO + saline group. (3) EPO treatment preserved the thickness of the retina.
Yamasaki et al. [74]	2005	Intervention study	RGCs isolated from neonatal Wistar rats	Assigned to three groups: (1) EPO (0.15, 0.5.0.15 U/mL) + vehicle; (2) EPO (0.15, 0.5.0.15 U/mL) + 1 mM glutamate; (3) EPO (0.15, 0.5.0.15 U/mL) + 10 mM glutamate. EPO was further tested in NO-induced toxicity: (1) Control; (2) EPO + NO-generating agent SNP (10, 100, 500 µM); (3) BDNF + NO-generating agent SNP (10, 100, 500 µM).	(1) RGCs in normal and ischemic retinas expressed EPOR protein. (2) EPO protected RGCs from glutamate-induced cytotoxicity, probably through its potential to prevent NO-induced cytotoxicity via reversal of Bcl-2 expression.
Zhong et al. [75]	2007	Intervention study	Total: 91 C57BL/6 J and 294 DBA/2 J mice	Assigned to three groups: (1) C57BL/6J mice; (2) DBA/2J mice; (3) DBA/2J mice + memantine. Assign to four groups: (1) DBA/2J +BSA (0.1%); (2) DBA/2J + EPO (3000 U/kg/wk); (3) DBA/2J + EPO (6000 U/kg/wk); (4) DBA/2J + EPO (12,000 U/kg/wk).	(1) Treatment with EPO at doses of 3000, 6000, and 12,000 U/kg body weight per week all prevented significant RGC loss. (2) The protective effect was similar to that of memantine.
Weishaupt et al. [76]	2004	Comparative study	Total: 25 adult Sprague Dawley rats	Optic nerve-transection mice, assigned to seven groups: (1) Axotomy + vehicle; (2) Axotomy + IVI EPO (0.5 U); (3) Axotomy + IVI EPO (1 U); (4) Axotomy + IVI EPO (2 U); (5) Axotomy + IVI EPO (4 U); (6) Axotomy + IVI EPO (8 U); (7) Axotomy + IVI EPO (2 U) + 0.1 mM Wortmannin.	(1) EPO prevented RGC death in the neurotrophic-factor-deprived and axotomized conditions. (2) EPO rescued axotomized RGCs mainly through the PI3K/Akt pathway. The neuroprotective effect was abolished through inhibition of PI3 kinase. (3) EPO protection was through suppression of activation of caspase-3.
Kilic et al. [77]	2005	Comparative study	Not mentioned	Transgenic tg21 mice (expressed human EPO in CNS under the control of the PDGF B chain promoter) and wild-type mice undergoing optic nerve transection	(1) EPO protected against RGC degeneration. (2) ERK1/2 and Akt signaling were increased, while JNK and caspase-3 signaling were decreased in axotomized tg21 mice. (3) EPO exerted its neuroprotection effects through the ERK-1/-2 pathway but not the Akt pathway.
King et al. [78]	2007	Comparative study	Total: 58 PVG hooded rats	(1) Optic nerve transection: -IVI EPO (5 U); -IVI EPO (10 U); -IVI EPO (25 U); -IVI EPO (50 U); -IVI PBS. (2) Optic nerve transection + peripheral nerve grafting: -IVI EPO (25 U); -IVI PBS.	(1) EPO administered intravitreally was both neuroprotective and neuroregenerative for axotomized RGCs in adult rats. (2) A small proportion of axons penetrated the transection site and regenerated up to 1 mm into the transected distal nerve.
Rex et al. [79]	2022	Comparative study	NA *	Mice with intracameral injection of microbeads, assigned to two groups: (1) IVI of EPO-R76E-loaded PLGA particles; (2) IVI of EPO-R76E-loaded PPS microspheres.	Both PLGA-EPO-R76E and PPS-EPO-R76E particles showed neuroprotective effects in a microbead-induced glaucoma mouse model through activation of the NRF2/ARE pathway.

IVI: intravitreal injection; EPO: erythropoietin; sEPOR: soluble erythropoietin receptor; I.P.: intraperitoneal injection; RGC: retinal ganglion cell; IOP: intraocular pressure; 2VO: bilateral common carotid artery occlusion; fVEP: flash visual evoked potentials; NO: nitric oxide; CNS: central nervous system; PDGF: platelet-derived growth factor; EPO-R76E: erythropoietin altered via arginine to glutamate at position 76; PLGA: poly (lactic-co-glycolic acid); PPS: poly (propylene sulfide). * The study was only available for review via abstract before this review was submitted.

**Table 2:** Summary of studies that evaluated EPO gene therapy for glaucoma.

Authors	Year	Study Design	Number of Animals	Intervention	Main Outcomes
Sullivan et al. [92]	2011	Comparative study	Total: 73 mice	Single IM for DBA/2J mice, assigned to five groups: (1) 3-month DBA/2J mice; (2) 10-month DBA/2J mice; (3) Control vector: rAAV2/5.CMV.eGFP; (4) Wild-type EPO vector: rAAV2/5.CMV.EPO; (5) Mutant EPO vector: rAAV2/5.CMV.EPOR76E.	(1) Both EPO vectors preserved axonal projections of the optic nerve. (2) Both EPO vectors retained visual function via flash visual evoked potentials. (3) The mutant EPO vector maintained hematocrits within normal limits, whereas the wild-type vector caused dangerously high hematocrit levels.
Bond et al. [93]	2016	Comparative study	Not mentioned	Single IM, assigned to two models. 1. C57BL/6J mice, microbead model: (1) Saline injection; (2) Control group: rAAV2/1.CMV. eGFP; (3) Mutant EPO vector: rAAV2/1.CMV. EPOR76E. 2. DBA/2J mouse group: (1) 3-month DBA/2J mice; (2) Control group: rAAV2/8.CMV. eGFP; (3) Mutant EPO vector: rAAV2/8.CMV. EPOR76E.	In both models, rAAV.EPOR76E: (1) Maintained normal RGC anterograde transport; (2) Preserved RGC axons; (3) Preserved flash visual evoked potentials; (4) Caused a hematocrit rise.
Hines-Beard et al. [94]	2016	Comparative study	Not mentioned	Single IM for DBA/2J mice, assigned to three groups: (1) 3-month DBA/2J mice; (2) Control vector: rAAV2/8.CMV.eGFP; (3) Mutant EPO vector: rAAV2/8.CMV.EPOR76E.	rAAV.EPOR76E: (1) Caused a rise in hematocrit; (2) Maintained normal RGC anterograde transport; (3) Preserved flash visual evoked potentials; (4) Decreased the Microglia number; (5) Lowered levels of proinflammatory cytokines/chemokines; (6) Increased levels of antioxidant enzymes.

IM: intramuscular injection; AAV: adeno-associated virus; CMV: cytomegalovirus; GFP: green fluorescent protein; RGC: retinal ganglion cell.

## Data Availability

Not applicable.

## Abbreviations

IOP	Intraocular pressure
EPO	Erythropoietin
HIF	Hypoxia-inducible factors
EPOR	Erythropoietin receptor
NMDA	N-methyl-D-aspartate
RGC	Retinal ganglion cell
BDNF	Brain-derived neurotrophic factor
MMP-9	Matrix metallopeptidase-9
ECM	Extracellular matrix
TNF-alpha	Tumor necrosis factor-alpha
NOS	Nitric oxide synthase
MRI	Magnetic resonance imaging
ET-1	Endothelin-1
NO	Nitric oxide
sGC	Soluble guanylyl cyclase
cGMP	Cyclic guanosine monophosphate
AMPA	α-amino-3-hydroxy-5-methyl-4-isoxazolepropionic acid
ROS	Reactive oxygen species
TrkB	Tropomyosin receptor kinase B
MAPK	Mitogen-activated protein kinases
PI3K	Phosphatidylinositol 3-kinase
PLC-ɣ	Phospholipase C-ɣ
EPOR2	Homogenous isoform of EPOR
EPO/BcR	Heterogeneous isoform of EPOR
Wnt	Wingless
JAK2	Janus kinase 2
STAT3/5	Signal transducer and activator of transcription 3/5
NF-κB	Nuclear factor-κB
Bcl-2	B-cell lymphoma 2
Bcl-XL	B-cell lymphoma-extra large
Akt	Protein kinase B
DNA	deoxyribonucleic acid
ICAM-1	Intercellular adhesion molecule-1
IL-1β	Interleukin-1 β
IL-6	Interleukin-6
IL-10	Interleukin-10
POAG	Primary open-angle glaucoma
ERG	Electroretinogram
TUNEL	Terminal deoxynucleotidyl transferase dUTP nick-end labeling
RAS	Rat sarcoma virus
RAF	Rapidly accelerated fibrosarcoma
ERK	Extracellular-signal-regulated kinase
EPOR76E	EPO altered arginine to glutamate at position 76
PLGA	Poly (lactic-co-glycolic acid)
PPS	Poly (propylene sulfide)
HSV	Herpes simplex virus
AAV9	Adeno-associated viral serotype 9
6-OHDA	6-hydroxydopamine
CMV	Cytomegalovirus
GFP	Green fluorescent protein
fVEP	Flash visual evoked potential
IL-12	Interleukin-12
IL-13	Interleukin-1
IL-17	Interleukin-17
CCL4	C-C motif chemokine ligand 4
CCL5	C-C motif chemokine ligand 5
Mcp-1	Monocyte chemoattractant protein-1
MSC	Mesenchymal stem cell
hWJMSC	Human mesenchymal stem cell from Wharton’s jelly
